# Recent Progress in Therapeutics and Materia Medica

**Published:** 1884-06-20

**Authors:** 


### RECENT PROGRESS IN THERAPEUTICS AND MATERIA MEDICA.

*Coco.*—Under the generic title of Erythroxylon, the last edition of the United States Pharmacopœia has offiicially recognized the Erythroxylon coco, which is known to have been used for ages by the natives of Peru and Bolivia as a stimulant, and especially to enable them to undergo protracted muscular exertion. The attention of the profession was first called by Wendell in 1853 to its usefulness as an accessory article of food, as a substitute for tea and coffee, since it produces, like them, effects of a gently stimulating character without possessing nourishing qualities of its own. A number of experimenters and clinical observers have confirmed this opinion and recommended its use in conditions of lowered vitality or extreme fatigue. It is probable that it also exerts some effect upon the kidneys, resulting in an increased flow of urine.

Dr. H. D. Hicks believes that the properties of this drug deserve to be better known to the profession. In a paper (New York Medical Journal, February 23), containing clinical records of three cases, its remarkable effects in relieving the sense of fatigue after extreme muscular exertion, and in sustaining the physical powers under unusual demands, and in weak heart, are well demonstrated. Dr. Hicks uses the remedy in his practice in order to prevent and relieve fatigue; to relieve pains in the back accompanied by the discharge of dark-colored urine; in dyspnœa due to weakness of muscles of respiration; for palpitation of heart due to dilatation or weakness of the heart-muscle without valvular lesions; mental exhaustion and low spirits; depression of nervous system following sexual excesses, sick-headache, etc. Finally he claims that it destroys the craving for alcohol, and that its habitual use as a part of the daily diet conduces to mental clearness and activity, freedom from fatigue, and sound sleep. These good effects were obtained from doses of half a drachm of the fluid extract several times daily.

*Action of the Opium-alkaloids.*—According to some recent researches by Von Schroder, conducted in Professor Schmiedeberg's laboratory in Strasburg, all the opium-alkaloids hitherto examined resemble opium in acting on the same part of the body, viz : the central nervous system. This conclusion holds good only for mammals, and must be qualified in regard to the frog, for in it narcotine, codeine, papaverine, and thebaine have also a paralizing action on the motor ganglia of the heart. These alkaloids agree with morphine not only in the organ they affect, but in the nature of their action. The symptoms may be divided into two stages : first, narcosis due to a paralytic action on the brain, followed by, second, tetanus due to increased irritability of the spinal cord. This agreement allows these alkaloids to be united with morphine into one group. Notwithstanding this qualitative agreement between the action of these alkaloids on the one hand and that of morphine on the other, there are considerable quantitative differences in the development and persistence of the narcotic and tetanic stages. The narcosis these alkaloids produce, unlike that of morphine, is not very deep, and quickly passes away; in the case of thebaine it occurs in the frog, as well as in mammals. The rapid development of the tetanic stage characterizes the action of this alkaloid. There is not, as in the case of morphine, a progressive paralysis gradually destroying the functions of the different parts of the brain; the action quickly extends over the whole brain, and remains slight while symptoms of irritation have already begun. This fact renders it advisable to break up the group of opium-alkaloids into two sub-groups, the first of which may be called the morphine group, characterized by the prominence of the narcotic stage, while in the other, which may be called the codeine group, the tetanic stage is more prominent, and the narcosis less so. The members of these groups may be arranged as follows, so that each subsequent member has a weaker narcotic, and in the codeine group has, at the same time, a stronger irritant action; in the morphine group oxydimorphine; in the codeine group papaverine, codeine, narcotine, thebaine. The codeine group contains also hydrocotarnine, laudanosine, and cryptopine; but at present we know too little about them to assign a place in the group to them with certainty. The same may be said of codethyline. The codeine group becomes closely allied by its last members with the strychnine group. The members of the codeine group should not be used therapeutically for their narcotic action. In the codeines produced from morphine by the addition of alcohol radicals such as codethyline, $C_{17}H_{18}NO_2(OC_2H_5)$, obtained from morphine by the introduction of ethyl, the narcotic action is diminished, whilst the conclusive action is increased in proportion to the number of atoms of hydrogen substituted by alcoholic radicals. In the alkaloids produced from morphine by oxidation (oxydimorphine and oxymorphine), their narcotic action is diminished, without the convulsant action being increased; narceine has no apparent physiological action.

*Effects of Arsenic, Mercury and Lead on the Spinal Cord.*—Dr. Podow found that in acute poisoning with arsenic, lead, and mercury, marked alterations occur in the spinal cord having the character of acute central myelitis. When the poisoning is comparatively slow, these changes are not limited to the gray substance alone, but affect the white substance also, and present the characters of diffuse myelitis. The peripheral nervous system is completely unaltered in rapid poisoning by these substances. The clinical nervous symptoms observed in rapid poisoning—convulsions, paralysis, pain, and anæsthesia—are explained by the alterations just described; but none of these symptoms can be explained by any peripheral nervous affection.

*Corrosive Sublimate in the Treatment of Diphtheria.*—In the Therapeutic Gazette, Dr. F. C. Herr publishes a communication strongly advocating the use of the corrosive chloride of mercury in the treatment of diphtheria. In an epidemic at Harrisburg it was administered to infants in doses of one-twelfth of a grain—every two hours for the first four doses, the interval then being increased to four hours—with excellent results. In one case two grains were given in the aggregate during the course of the treatment without bad effects. The original states that ten grains were given; but Dr. Herr informed us that this was a misprint, and that the amount given was that which we have just stated.

*A New Use for Santonin.*—A case under the care of Dr. N. Anderson (Lancet, November 10), suffering with lumbricoid worms, reported that, as the result of his treatment, he had been relieved of his worms, and also that a long-standing gleet had ceased. The reporter thereupon recommends santonin for gleet, five grains rubbed up with an equal quantity of sugar of milk, to be taken twice a day in milk. It is possible, in the case reported, that the effect of santonin upon the gleet was due to a secondary, and not to a direct or primary, action; however, there need be no difficulty about finding suitable subjects to try it upon. *Fiat experimentum in corpore vile.—Phila. Med. Times.*

